# Investigation of Plasmids Among Clinical *Staphylococcus aureus* and *Staphylococcus haemolyticus* Isolates From Egypt

**DOI:** 10.3389/fmicb.2021.659116

**Published:** 2021-06-04

**Authors:** Carine R. Mores, Cesar Montelongo, Catherine Putonti, Alan J. Wolfe, Alaa Abouelfetouh

**Affiliations:** ^1^Department of Microbiology and Immunology, Stritch School of Medicine, Loyola University Chicago, Chicago, IL, United States; ^2^Bioinformatics Program, Loyola University Chicago, Chicago, IL, United States; ^3^Department of Biology, Loyola University Chicago, Chicago, IL, United States; ^4^Department of Microbiology and Immunology, Faculty of Pharmacy, Alexandria University, Alexandria, Egypt; ^5^Department of Microbiology and Immunology, Faculty of Pharmacy, Alalamein University, Alalamein, Egypt

**Keywords:** antibiotic resistance, *Staphylococcus*, plasmids, incompatibility group, Rep, horizontal gene transfer

## Abstract

Staphylococci can cause a wide array of infections that can be life threatening. These infections become more deadly when the isolates are antibiotic resistant and thus harder to treat. Many resistance determinants are plasmid-mediated; however, staphylococcal plasmids have not yet been fully characterized. In particular, plasmids and their contributions to antibiotic resistance have not been investigated within the Arab states, where antibiotic use is not universally regulated. Here, we characterized the putative plasmid content among 56 *Staphylococcus aureus* and 10 *Staphylococcus haemolyticus* clinical isolates from Alexandria, Egypt. Putative plasmid sequences were detected in over half of our collection. In total, we identified 72 putative plasmid sequences in 27 *S. aureus* and 1 *S. haemolyticus* isolates. While these isolates typically carried one or two plasmids, we identified one isolate—*S. aureus* AA53—with 11 putative plasmids. The plasmid sequences most frequently encoded a Rep_1, RepL, or PriCT_1 type replication protein. As expected, antibiotic resistance genes were widespread among the identified plasmid sequences. Related plasmids were identified amongst our clinical isolates; homologous plasmids present in multiple isolates clustered into 11 groups based upon sequence similarity. Plasmids from the same cluster often shared antibiotic resistance genes, including *blaZ*, which is associated with β-lactam resistance. Our analyses suggest that plasmids are a key factor in the pathology and epidemiology of *S. aureus* in Egypt. A better characterization of plasmids and the role they contribute to the success of Staphylococci as pathogens will guide the design of effective control strategies to limit their spread.

## Introduction

Staphylococci are commensal bacteria in humans and animals with the ability to cause serious infections. Prime among the Staphylococci is *Staphylococcus aureus* with pathologies that range from superficial skin lesions to bacteremia, pneumonia, and endocarditis, among others (Tong et al., 2015). *S. aureus* is a major challenge in both the hospital and community settings, with treatment made more difficult given its ability to rapidly acquire antibiotic resistance (Zetola et al., 2005; Grundmann et al., 2006; Foster, 2017). The archetypal and most common antibiotic resistant examples are the Methicillin-resistant *S. aureus* (MRSA) strains (Foster, 2017), which are usually resistant to most β-lactam antibiotics (Hill-Cawthorne et al., 2014). Strains of Coagulase-Negative Staphylococci (CoNS), including *Staphylococcus epidermidis* and *Staphylococcus haemolyticus*, also have acquired antibiotic resistance (Becker et al., 2014). For example, over the past 40 years, Methicillin-resistant *S. haemolyticus* (MRSH) strains have become more prevalent (Ferreira et al., 2002; Bouchami et al., 2011; Becker et al., 2014; Xu et al., 2018; Al-Tamimi et al., 2020). To treat MRSA and MRSH, clinicians must resort to different classes of antibiotics, but further acquisition of antibiotic resistance by Methicillin-resistant strains is a major problem (Hill-Cawthorne et al., 2014). In Egypt, antibiotic use is not regulated, and most antimicrobial agents are available without the need for a prescription. This unregulated availability increases selective pressure that leads to development of antibiotic resistance. Thus, antibiotic therapy is often inadequate, a problem compounded by the use of the wrong antibiotic and both inappropriate dosage and duration. Consequently, Egypt has seen alarming increases in antibiotic resistance and MRSA/MRSH prevalence (Borg et al., 2007; Falagas et al., 2013; Abouelfetouh, 2017; Mashaly and El-Mahdy, 2017).

Horizontal gene transfer is a major source for acquisition of antibiotic resistance genes in Staphylococci, facilitated by phage transduction and plasmid conjugation (McCarthy et al., 2014). Plasmids can be reservoirs and vectors for antibiotic resistance, virulence, and fitness genes, even in the absence of selective pressure (Kwong et al., 2017). Horizontal transfer of plasmids can occur not only between Staphylococcal strains of the same species but also between species, with CoNS believed to act as reservoirs for antibiotic resistance genes (Otto, 2013). Moreover, recent evidence suggests that plasmids also can be exchanged between Staphylococci and the distantly related Enterococci (Acman et al., 2020). Data on the diversity of plasmid content of Staphylococci is limited (Borg et al., 2007; Abouelfetouh, 2017; Abouelfetouh et al., 2017; Kwong et al., 2017), but it is known that Staphylococcal plasmids can range in size from 1 to 60 kbp and that they can carry resistance genes (or gene clusters) (Kwong et al., 2017). Clinical isolates often contain one or more multi-resistance plasmids (Shearer et al., 2011). Thus, plasmids are key to the epidemiology of Staphylococci, contributing to the rapid spread of antibiotic resistance, and play a major role in determining clinical outcomes (Hisatsune et al., 2013; Shahkarami et al., 2014). While over 1,000 *S. aureus* plasmid sequences and 15 *S. haemolyticus* plasmid sequences are publicly available in resources such as PLSDB (Galata et al., 2019), just 6 *S. aureus* and no *S. haemolyticus* plasmids are annotated as collected from the Arab states. This knowledge gap motivated our investigation of plasmids from this region, and we hypothesize that plasmids in Egyptian Staphylococcal strains encode for antibiotic resistance.

Large plasmids in bacteria can be classified by their incompatibility groups, as plasmids with similar replication initiation (Rep) proteins cannot be stably maintained in the absence of selective pressure (Jensen et al., 2010; McCarthy and Lindsay, 2012). To date, 7 distinct types of Rep proteins and 15 incompatibility groups have been identified for Staphylococcal plasmids (Kwong et al., 2017). Accordingly, Staphylococcal plasmids can be classified using the sequence of their Rep genes (Jensen et al., 2010). Antibiotic resistance genes may be linked to Rep families; for example, tetracycline resistance has been associated with the *rep7* gene and chloramphenicol resistance with the *rep7*, *rep13*, and *rep27* genes (McCarthy and Lindsay, 2012). Currently, there are limited data on the Rep families present in circulating Staphylococcal isolates, including those from Egypt. Knowledge of the plasmid Rep families present in these clinical isolates could give us insight into Staphylococcal plasmid biology and thus help to better understand Methicillin-resistant strains.

In this study, we utilized genomics to (i) catalog the Rep family classification in the plasmids of Egyptian clinical isolates of *S. aureus* and *S. haemolyticus*, (ii) profile the antibiotic resistance genes present in these plasmids, and (iii) assess the prevalence of plasmid-borne antibiotic resistance in Egyptian MRSA isolates. We report on 72 plasmids present in 27 *S. aureus* and 1 *S. haemolyticus* isolates from Egypt. We also report on the antibiotic resistance genes in these plasmids, including those from MRSA and MRSH isolates. We furthermore provide bioinformatic and genetic context for these plasmid sequences. We found that the plasmids in Egyptian isolates of *S. aureus* and *S. haemolyticus* are diverse and include antibiotic resistance genes for antibiotics used clinically, indicating the potential for plasmids to act as gene reservoirs and thus facilitate the rapid horizontal transfer of antibiotic resistance in Egypt.

## Materials and Methods

### Identifying Plasmid Sequences

Raw sequencing reads for the 56 *S. aureus* and 10 *S. haemolyticus* genomes were previously sequenced, annotated, and typed by our group (Montelongo et al., 2021). Here, the raw reads were assembled using plasmidSPAdes (v3.9.0) (Antipov et al., 2016). Plasmid sequences with a length greater than 1,000 bp were selected for further analysis. Each putative plasmid sequence was queried via megablast against the nr/nt database (April 2020). The best hit to a complete plasmid sequence record for each sequence was identified and recorded. PlasmidFinder (v2.1) hosted by the Center for Genomic Epidemiology was queried to identify replicon sequences (Carattoli et al., 2014). BLAST and PlasmidFinder results were considered together to distinguish putative plasmids from other sequences identified by plasmidSPAdes (e.g., bacteriophage). To verify even coverage, reads were mapped to plasmid sequences using the Bowtie2 (v2.3.2) (Langmead and Salzberg, 2012) plug-in through Geneious Prime v2019.1.1 (Biomatters Ltd., Auckland, NZ). To estimate plasmid copy numbers, genome and plasmid coverages were calculated using BBMap v38.47^1^.

### Annotating Plasmid Sequences

Putative plasmid sequences were annotated using PATRIC v3.6.3 (Brettin et al., 2015; Wattam et al., 2017). Putative plasmid sequences also were examined for antibiotic resistance using ResFinder (v3.2) (Zankari et al., 2012), using default parameters (%ID threshold 90%, selected minimum length 60%).

### Clustering Similar Plasmids

Putative plasmid sequences were clustered using the USEARCH algorithm (v11.0.667) (Edgar, 2010) with the “cluster_fast” option for id = 0.8 to both strands. Each cluster was aligned using MAFFT v7.388 (Katoh and Standley, 2013) and Mauve (Darling et al., 2004) through Geneious Prime and manually inspected. BLAST hits were referenced to verify that plasmids within the same cluster exhibited similarity to related (if not the same) plasmid sequence in the nr/nt database. Plasmid alignments were generated using BLAST Ring Image Generator v0.95 (Alikhan et al., 2011).

### Phylogenomic Tree of *S. aureus* Strains

Anvi’o (Eren et al., 2015) was used to identify the single copy number genes amongst the *S. aureus* genome sequences. A phylogenomic tree was created using concatenated amino acid sequences for these single copy number genes. These concatenated sequences were aligned using MAFFT v7.388 (Katoh and Standley, 2013) through Geneious Prime (Biomatters Ltd., Auckland, New Zealand). The tree was derived using FastTree v2 (Price et al., 2010) through Geneious Prime and visualized by iTOL v5.6.1 (Letunic and Bork, 2019).

## Results

Upon the examination of 56 *S. aureus* and 10 *S. haemolyticus* genomes of Egyptian clinical isolates, we identified 72 putative plasmid sequences in 28 strains: 27 strains of *S. aureus* and 1 strain of *S. haemolyticus*. From our previous work (Montelongo et al., 2021), we know that 24 of these isolates are *mecA*-positive and represent 11 different MLSTs and 2 unknown MLSTs. While 10 of the strains harbored just a single putative plasmid sequence, one strain—AA53—contained 11 (Table 1). The copy number of the plasmid sequences ranged from ∼1 to >300, determined by comparing the coverage of each individual plasmid sequence relative to the average coverage for the assembly (Supplementary Table 1). 50 of the 72 putative plasmid sequences contained replicon proteins identified by PlasmidFinder. Table 2 summarizes these results. The majority of the strains (85%) contained plasmid sequences associated with rep16 (*n* = 10), rep20 (*n* = 9), or rep21 (*n* = 9). The replicons rep22, rep24a, and rep39 were each unique to a single isolate. As expected because of incompatibility, no strain was found to have more than one plasmid for a given replicon protein. Putative plasmid sequences containing a replicon protein frequently showed sequence identity to a *Staphylococcus* plasmid sequence (Supplementary Table 2).

**TABLE 1:** List of Egyptian *S. aureus* and *S. haemolyticus* strains harboring plasmids.

**Strain**	**MLST**	**Methicillin resistance**	**GenBank WGS master accession**	**# Plasmids**
*S. aureus* AA1	ST-1	*mecA* +ve	JAEOUR000000000	2
*S. aureus* AA2	ST-80	*mecA* +ve	JAEOUZ000000000	5
*S. aureus* AA3	ST-80	*mecA* +ve	JAEOVE000000000	5
*S. aureus* AA4	ST-80	*mecA* +ve	JAEOVM000000000	5
*S. aureus* AA5	ST-22	*mecA* +ve	JAEOVQ000000000	1
*S. aureus* AA6	ST-97	*mecA* +ve	JAEOVX000000000	1
*S. aureus* AA8	ST-97	*mecA* +ve	JAEOWM000000000	1
*S. aureus* AA13	ST-239	*mecA* +ve	JAEOUV000000000	1
*S. aureus* AA17	ST-152	*mecA* +ve	JAEOUX000000000	3
*S. aureus* AA29	ST-239	*mecA* +ve	JAEOVD000000000	2
*S. aureus* AA30	ST-6	*mecA* +ve	JAEOVF000000000	1
*S. aureus* AA32	Unknown^a^	*mecA* +ve	JAEOVH000000000	1
*S. aureus* AA35	Unknown	*mecA* +ve	JAEOVJ000000000	4
*S. aureus* AA36	ST-97	*mecA* +ve	JAEOVK000000000	1
*S. aureus* AA41	ST-1482	*mecA* -ve	JAEOVN000000000	2
*S. haemolyticus* AA42	ST-30	*mecA* +ve	JAEOWY000000000	2
*S. aureus* AA45	ST-80	*mecA* +ve	JAEOVO000000000	4
*S. aureus* AA51	ST-1	*mecA* +ve	JAEOVR000000000	2
*S. aureus* AA53	ST-88	*mecA* +ve	JAEOVT000000000	11
*S. aureus* AA57	ST-239	*mecA* +ve	JAEOVV000000000	2
*S. aureus* AA59	ST-1	*mecA* -ve	JAEOVW000000000	1
*S. aureus* AA63	ST-239	*mecA* +ve	JAEOWB000000000	3
*S. aureus* AA64	ST-239	*mecA* +ve	JAEOWC000000000	3
*S. aureus* AA65	ST-1	*mecA* -ve	JAEOWD000000000	3
*S. aureus* AA68	ST-1	*mecA* -ve	JAEOWF000000000	1
*S. aureus* AA69	ST-1	*mecA* +ve	JAEOWG000000000	2
*S. aureus* AA70	ST-5	*mecA* +ve	JAEOWH000000000	1
*S. aureus AA78*	ST-1	*mecA* +ve	JAEOWK000000000	2

*^*a*^Incomplete alignment.*

**TABLE 2:** Number and type of replicons identified by PlasmidFinder for plasmid sequences in the Egyptian *S. aureus* strains and *S. haemolyticus* strain.

**Strain**	**# Replicons**	**Replicon protein**								
		**5a^a^**	**7a^b^**	**10^c^**	**16^d^**	**20^e^**	**21^f^**	**22^f^**	**24a^e^**	**39^e^**
*S. aureus* AA1	2			+	+					
*S. aureus* AA2	1					+				
*S. aureus* AA3	1					+				
*S. aureus* AA4	2					+	+			
*S. aureus* AA5	1	+								
*S. aureus* AA6	1					+				
*S. aureus* AA8	1					+				
*S. aureus* AA13	1			+						
*S. aureus* AA17	3		+	+	+					
*S. aureus* AA29	2		+	+						
*S. aureus* AA30	1				+					
*S. aureus* AA32	1	+								
*S. aureus* AA35	4		+	+		+	+			
*S. aureus* AA36	1					+				
*S. aureus* AA41	1				+					
*S. aureus* AA45	1					+				
*S. aureus* AA51	1				+					
*S. aureus* AA53	6	+	+	+		+	+		+	
*S. aureus* AA57	2		+				+			
*S. aureus* AA59	1				+					
*S. aureus* AA63	3		+	+			+			
*S. aureus* AA64	3		+	+			+			
*S. aureus* AA65	3			+	+		+			
*S. aureus* AA68	1				+					
*S. aureus* AA69	1				+					
*S. aureus* AA70	1						+			
*S. aureus* AA78	2				+			+		
*S. haemolyticus* AA42	2						+			+

*“+” indicates the presence of a plasmid. Replication Protein Types: ^*a*^Rep_3, ^*b*^Rep_trans, ^*c*^RepL, ^*d*^PriCT_1, ^*e*^RepA_N, ^*f*^Rep_1.*

**FIGURE 1:**
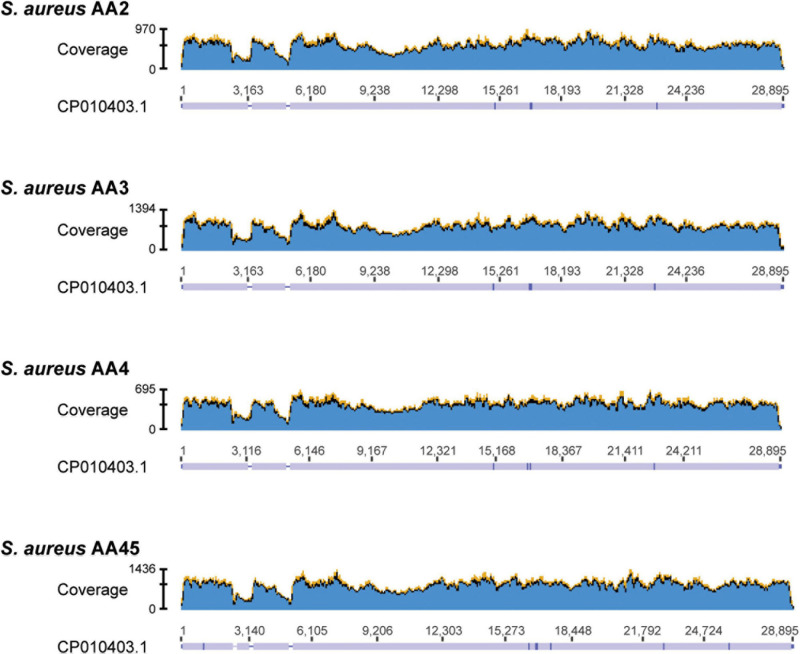
Read coverage maps for the 4 *S. aureus* strains containing contigs showing homology to plasmid pGR2A (CP010403.1). Raw reads from each of the 4 strains were mapped to the pGR2A sequence and their coverage is shown in the graphs. Below each graph, the consensus sequence is shown. Regions covered appear in lavender. Breaks in this coverage (lavender bar) indicates regions where no reads mapped.

To ascertain if the 22 putative plasmid sequences that did not contain a replicon protein likely represented a plasmid, we referred to the BLAST hits (Supplementary Table 2). These all showed greatest sequence similarity (in some cases, even 100% sequence identity) to a previously reported Staphylococcal plasmid sequence, often one that was significantly longer than our putative plasmid sequence. Of note are the putative plasmid sequences within *S. aureus* strains AA2, AA3, AA4, and AA45. Each of these strains contained three or four sequences that showed sequence similarity to the same *S. aureus* plasmid sequence, pGR2A (Accession no. CP010403). For each of these strains, one sequence had a recognizable rep20 replicon. We downloaded the pGR2A record and mapped the raw reads for strains AA2, AA3, AA4, and AA45 to the pGR2A sequence. While the reads mapped to the plasmid sequence, there were two or three regions of low coverage (Figure 1), suggesting that either the plasmid within these strains: (1) has undergone reassortment or expansion, differentiating it from pGR2A; (2) has split into separate extrachromosomal DNAs; (3) contains a region that was not sequenced by the Illumina short-read technology; and/or integrated into the genome. The pGR2A-like plasmid sequences accounted for 14 of the 22 putative plasmid sequences without a recognized replicon protein. Thus, there are just eight putative plasmid sequences in our data set for which a replicon protein was not identified.

**FIGURE 2:**
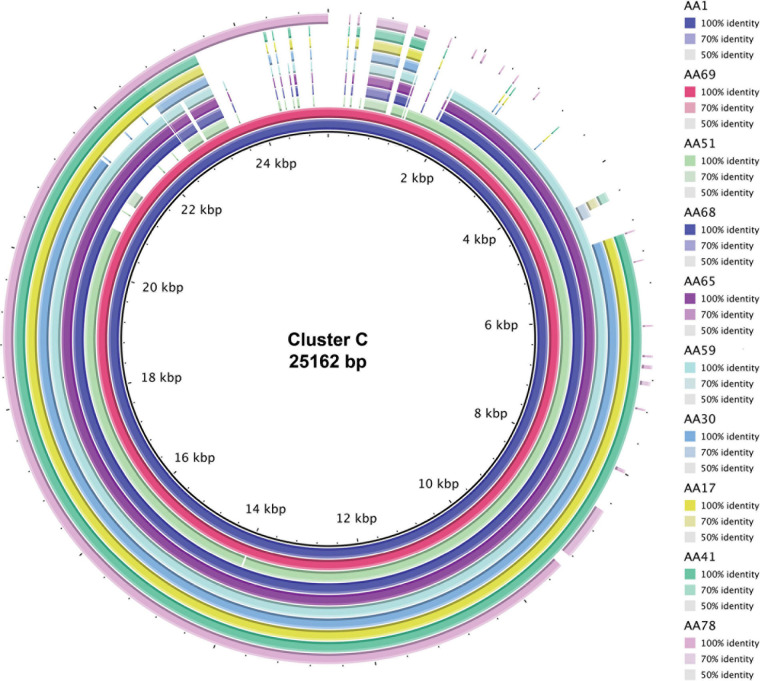
Sequence alignment of the 10 plasmid sequences grouped into Cluster C. The legend indicates the color for the plasmid sequence with the darker tones indicating 100% sequence identity and lighter tones indicative of less sequence identity. Gaps in the alignment signify regions that are not found within the plasmid sequence. The total alignment is 25,162 bp.

**FIGURE 3:**
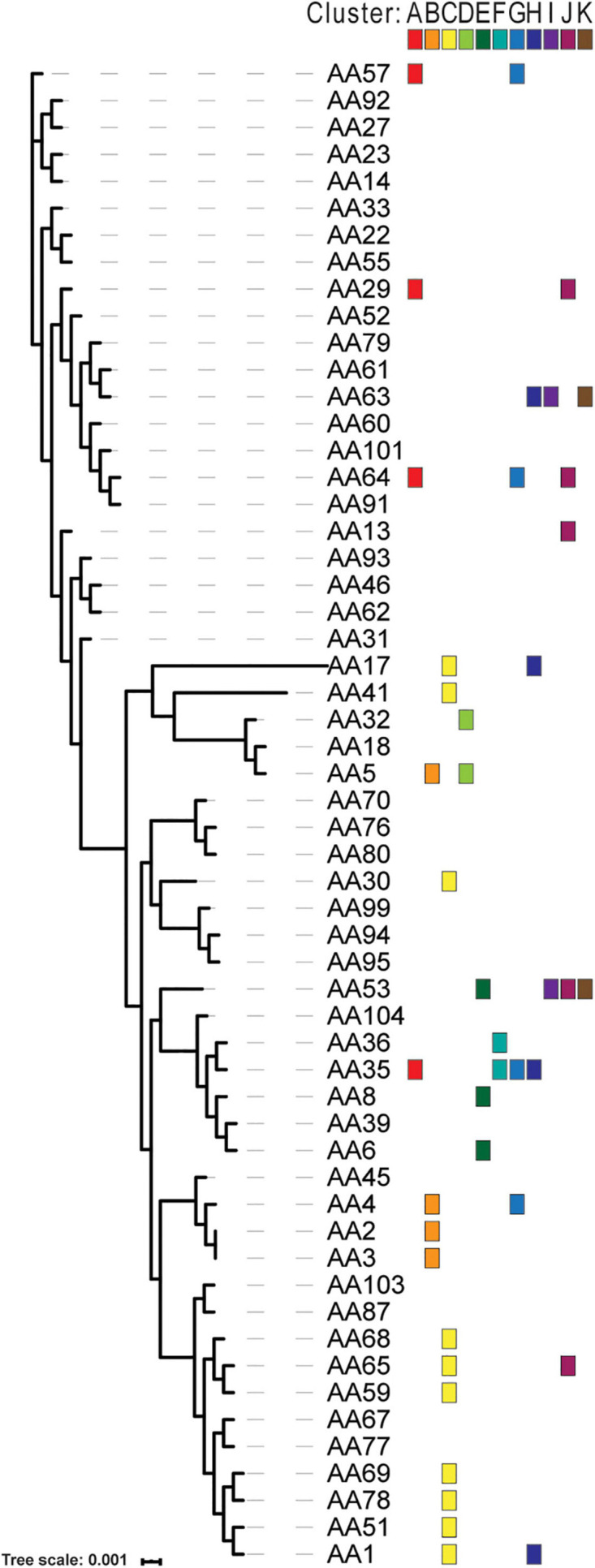
Distribution of plasmid clusters across phylogenomic diversity of Egyptian *S. aureus* strains. The phylogenomic tree for the isolates was generated based upon the concatenated amino acid sequences of the single copy number genes in the core genome. The tree scale indicates the sequence divergence between the core genome sequence. The color blocks show the presence of a plasmid belonging to 1 of the 11 clusters.

To identify homologous plasmids found in more than one isolate, all 72 putative plasmid sequences were clustered. In total, we identified 11 clusters while 16 plasmids did not show any sequence similarity to another plasmid in our data set. These 16 unclustered plasmids, however, did exhibit sequence similarity to plasmid sequences deposited in GenBank (Supplementary Table 2). This included the two plasmids within the *S. haemolyticus* A42 strain. Table 3 lists details about these clusters. The largest clusters, plasmid Cluster C and plasmid Cluster J, contained a plasmid sequence found in 10 and 5 different strains, respectively. In some cases, all members of the cluster showed greatest homology to the same GenBank plasmid record, for instance Cluster A (*n* = 4), Cluster B (*n* = 4), Cluster D (*n* = 2), Cluster E (*n* = 2), and Cluster K (*n* = 2). In other cases, members of the cluster exhibited greatest sequence similarity to different GenBank plasmid records, reflecting the differences between the homologous plasmids, as well as hinting at diversity in the plasmid group amongst strains of *S. aureus*. Figure 2 shows that instances of gene acquisition/loss can be detected between homologous plasmids in a cluster. For some clusters, the plasmids are carried by closely related *S. aureus* strains, while other clusters include plasmids found in strains that span the genomic diversity of the Egyptian isolates (Figure 3).

**TABLE 3:** Clusters of plasmids based on nucleotide sequence similarity within the Egyptian *S. aureus* strains.

**Cluster ID (replicon)**	**Cluster details**				**Closest relative (by BLAST homology)**		
	**Strains**	**Avg. length (bp)**	**%GC**	**% Pairwise identity**	**Description**	**Accession #**	**Length (bp)**
A (rep7a)	AA29, AA35, AA57, AA64	3,909	29.3	99.5	Unnamed3	CP033985	3,785
B (rep20)	AA2, AA3, AA4, AA45	27,938	29.1	100	pGR2A	CP010403	28,895
C (rep16)	AA17	20,970	28.7	80.7	pPS00087.1A.1	CP022721	20,730
	AA30				pl1_M2024	CP047022	19,760
	AA41				pWBG757	GQ900397	20,730
	AA1, AA59, AA65, AA68, AA69, AA78				pWBG750	GQ900392	20,653
	AA51				p18809-P04	CP002146	28,404
D (rep5a)	AA5, AA32	34,114	30.3	100	pIT4-R	CP028471	34,104
E (rep20)	AA6, AA8, AA53	16,610	28.1	99.2	pM121	CP007671	20,409
F (rep20)	AA35	21,196	28.4	80	Unnamed1	CP030394	25,071
	AA36				Unnamed1	CP030413	20,403
G (rep21)	AA35	3,112	29	99.5	pV605	CP013960	43,115
	AA4				SAP104A	GQ900450	3,011
	AA57, AA64				pV605	CP013960	43,115
H (rep10)	AA1	2,560	30.8	96.8	Unnamed1	CP029648	2,473
	AA17				pSau-2716Lar	MH423311	3,267
	AA35				Unnamed	CP047816	2,473
	AA63				Unnamed2	CP047819	2,477
I (rep21)	AA53	3,123	29.1	98.8	3	LR130517	3,011
	AA63				Unnamed1	CP034006	3,011
J (rep10)	AA13	2,556	31	98.9	pUSA05-1-SUR24	CP014446	2,415
	AA29				pSR265	MN251859	2,413
	AA53				p19321-P01	CP002148	2,473
	AA64				SAP078B	GQ900431	2,415
	AA65				pSR241	MN251858	2,402
K (rep7a)	AA53, AA63	3,901	29.6	98.8	pOC160-2	LC012933	3,788

We annotated the plasmid sequences using the web service PATRIC (Supplementary Table 3). 38.6% of the coding regions within the 72 plasmid sequences are annotated as hypothetical proteins. PATRIC did identify resistances to metals, e.g., cadmium, copper, cobalt/zinc/cadmium, lead, mercury, and zinc, and antibiotics. Furthermore, the plasmid from *S. aureus* AA70 also encodes for exotoxin proteins. To further investigate antibiotic resistance genes, each plasmid sequence was examined with the tool ResFinder. Table 4 summarizes the antibiotic resistance determinants predicted by ResFinder, and full results can be found in Supplementary Table 4. 20 of the 27 (74%) *S. aureus* strains included a putative plasmid encoding *blaZ*, which is associated with β-lactam resistance. These included all four of the *mecA*-negative *S. aureus* strains (AA41, AA59, AA65, and AA68). *S. aureus* AA69 and AA41 were the only strains carrying a plasmid encoding aminoglycoside resistance. The one plasmid-carrying *S. haemolyticus* strain (AA42) did not encode *blaZ*; however, it carried plasmids that encode for lincosamide resistance, macrolide resistance, and streptogramin B resistance. This strain is the only one identified in this study that carries a plasmid that encodes for the *lnu(A)* gene, which is associated with lincosamide resistance.

**TABLE 4:** Resistance genes carried on Egyptian *S. aureus* and *S. haemolyticus* plasmids.

**Predicted phenotype**	**Resistance gene**	**Strains**
Aminoglycoside resistance	*aac(6′)-aph(2”)*	AA69
	*ant(6)-Ia*	AA41
	*aph(3′)-III*	AA41
Beta-lactam resistance	*blaZ*	AA1, AA2, AA3, AA4, AA6, AA8, AA17, AA30, AA35, AA36, AA41, AA45, AA51, AA53, AA59, AA65, AA68, AA69, AA70, AA78
Fusidic acid resistance	*fusB*	AA2, AA3, AA4, AA45
Lincosamide resistance	*lnu(A)*	AA42
Macrolide resistance	*erm(C)*	AA1, AA1, AA13, AA17, AA29, AA35, AA53, AA63, AA63, AA64, AA65
	*mph(C)*	AA41, AA42
Macrolide, Lincosamide, and Streptogramin B resistance	*msr(A)*	AA41, AA42
Phenicol resistance	*cat(pC221)*	AA29, AA35, AA35, AA35, AA53, AA57, AA63, AA64, AA64, AA64
Tetracycline resistance	*tet(K)*	AA2, AA3, AA4, AA17, AA45
	*tet(L)*	AA1, AA51, AA59, AA65, AA68, AA69, AA78

Whereas 48.6% of the plasmids carried a single antibiotic resistance gene, others carried multiple antibiotic resistance genes. We therefore examined the plasmid sequences for resistance co-occurrence. Strains harboring plasmids encoding for fusidic acid resistance (*fusB*) (*n* = 4) also harbored distinct plasmids that encoded for tetracycline resistance [*tet(K)*] (*n* = 5) (*r* = 0.9). Furthermore, all four strains with plasmids carrying both *fusB* and *tet(K*) were MLST-80. Plasmids encoding for *tet(L)* were only harbored by strains of MLST-1. However, this association between *tet(L)* and MLST-1 was not observed more broadly; publicly available MLST-1 genomes (Supplementary Table 5) were queried for the *tet(L)* sequence. Only 8 of the 112 strains queried also coded for *tet(L)*. Interestingly, six of these were Egyptian MRSA isolates (Supplementary Table 5).

## Discussion

Plasmids are key reservoirs for genetic content in Staphylococci and allow the rapid propagation of antibiotic resistance (Jensen et al., 2010; McCarthy and Lindsay, 2012). Here, we screened the genomes of 56 clinical isolates of *S. aureus* and 10 clinical isolates of *S. haemolyticus* previously sequenced by our group and found 72 putative plasmid sequences (Table 1). We identified replicon proteins in 50 of the 72 plasmids. Within the *S. aureus* strains, eight different replicon proteins were detected, representative of six of the seven Staphylococcal replicon protein types, and the *S. haemolyticus* isolate included a plasmid with an additional replicon protein not found in the *S. aureus* strains, rep39 (Table 2). This particular plasmid (rep39) was homologous to pLNU4, a plasmid found in an *S. chromogenes* isolate that was associated with bovine mastitis, and can be acquired by *S. aureus* via transformation (Lüthje et al., 2007). For strains containing more than 1 plasmid, 11 different combinations of the replicon proteins were found. Strains containing plasmids with rep7a and rep10 were most common (*n* = 6); whereas one strain, *S. aureus* AA13, contained just a single rep10 plasmid, no strains contained a single rep7a plasmid. Rep sites were not identified in eight of the putative plasmids, although their sequence reads had homology to plasmid sequences. These eight could represent partial plasmid sequences or may rely on vertical transfer of plasmids, as is the case of smaller plasmids with high copy number (Kwong et al., 2017). However, based on both plasmid copy number (Supplementary Table 1) and BLAST hits (Supplementary Table 2), we believe that these eight sequences are in fact partial plasmid sequences.

*S. aureus* strains AA2, AA3, AA4, and AA45 had putative plasmid sequences that showed sequence similarity to the *S. aureus* plasmid pGR2A (Accession no. CP010403). The pGR2A plasmid codes a unique *bla* system that can suppress oxacillin resistance in MRSA strains (Sabat et al., 2015). The non-functional BlaR1 from pGR2A does not cleave the *mecA* repressor BlaI; thus, *mecA* is expressed inefficiently, resulting in a phenotype of penicillin resistance, but oxacillin susceptibility (Sabat et al., 2015). It must be noted that all four strains with plasmid sequences homologous to pGR2A were *mecA*-positive strains (Tables 1, 3). In the clinical setting, oxacillin-susceptible *mecA*-positive *S. aureus* may pose a challenge due to possible misdiagnosis as a typical MRSA (Ikonomidis et al., 2008; Sabat et al., 2015).

In addition to classifying plasmids by their Rep genes, we analyzed plasmid relatedness by clustering putative plasmid-coding regions based on sequence homology (Table 3). We identified 11 plasmid clusters among the *S. aureus* strains; the two *S. haemolyticus* plasmid sequences did not resemble any of the *S. aureus* plasmids. Members of a cluster could have homology to different GenBank plasmid records (C, J), or all could share the same GenBank plasmid record (A, B, D, E, K). The former likely indicates homology of non-identical plasmids and the acquisition/loss of content as indicated by cluster C (Figure 2), while the latter points to multiple *S. aureus* isolates possessing the same plasmid, potentially due to a shared selective pressure (Lüthje et al., 2007; Kwong et al., 2017). Our results found that Rep genes are not exclusive to a single cluster, concurring with prior findings of frequent gene exchange amongst Staphylococcal plasmids (see review; Kwong et al., 2017). Strains harboring plasmids from the same cluster often shared antibiotic resistance genes, e.g., macrolide resistance in Cluster A plasmids, fusidic acid resistance in Cluster B, and beta-lactam resistance in Cluster C. Thus, while replicon proteins can provide insight into plasmid incompatibilities, they have limited epidemiological information compared to the cluster to which a plasmid belongs.

Of the 28 isolates in which we identified putative plasmid sequences, 24 (86%) were *mecA*-positive in addition to widespread presence of resistance genes in their plasmid sequences (Table 4). In the clinical setting, the main classes of drugs used against *Staphylococcus* are those that target the cell wall (e.g., β-lactam antibiotics, glycopeptides), ribosome (e.g., tetracyclines, aminoglycosides, macrolides), and nucleic acid biosynthesis (fluoroquinolones and sulfamethoxazole/trimethoprim) (Foster, 2017). Resistance to these antibiotic classes is a critical concern, especially in the acquisition of multiple resistances (Grundmann et al., 2006; Falagas et al., 2013). There have been reports of over-the-counter antibiotic use in Egypt that could select for resistance to key antibiotics, e.g., penicillins and cephalosporins (Abouelfetouh, 2017; Abouelfetouh et al., 2017). The most common antibiotic resistances in the plasmid sequences were those to macrolides, tetracyclines, fusidic acid, and aminoglycosides, and 20 of our *Staphylococcus* putative plasmid sequences had genes linked to β-lactam antibiotics resistance (Table 4). While tetracycline resistance can be plasmid- or chromosome-mediated, macrolide resistance is almost exclusively encoded by mobile elements in Staphylococci. Nine different Egyptian *S. aureus* strains carried plasmids with the 23S rRNA methylase *erm(C)* (Table 4). Previous studies have found that *erm(C)* is the most abundant gene conferring resistance to macrolides, as well as to lincosamides and streptogramin B compounds (see review; Feßler et al., 2018). *mph(C)*, found in *S. aureus* AA41 and *S. haemolyticus* AA42, mediates resistance to only macrolides, whereas *lnu(A)*, found only in *S. haemolyticus* AA42, encodes only lincosamide resistance. In addition, our annotation indicated resistance genes for chloramphenicol, clindamycin, and sulfamethoxazole/trimethoprim (Supplementary Table 3). The presence of these antibiotic resistance genes can greatly limit the antibiotic options to treat these clinical isolates (Abouelfetouh, 2017; Foster, 2017; Vestergaard et al., 2019). Of utmost concern is that multiple resistances could be disseminated via plasmids, especially in MRSA (McCarthy and Lindsay, 2012).

We did not identify genes involved in conjugation in our isolates (Supplementary Table 3). It has been noted that *S. aureus* may rely on vertical transmission of its plasmids, while conjugation genes are relatively rare (McCarthy and Lindsay, 2012). The distribution of plasmids belonging to the same cluster across the Egyptian *S. aureus* isolates suggests both vertical and horizontal transfer (Figure 3). Horizontal acquisition of the plasmid in our isolates could occur via natural competence or by conjugation systems not yet characterized and therefore not present in our annotation references (Morikawa et al., 2012). It must be highlighted that many of the plasmid sequences presented in these Egyptian isolates were homologous to GenBank entries for plasmids that have not been well-characterized (Table 3); from 37 plasmids homologous to our sequences, only seven had a publication associated with their entry. This underscores the general lack of *S. aureus* plasmid knowledge. Furthermore, 38.6% of the genes annotated within our plasmids are characterized as hypothetical proteins. Their function and/or the possible genetic advantage that these genes provide the bacterial strains remains an open question. The presence of genes for multiple antibiotic resistance in uncharacterized plasmids indicates not just the relevance of plasmids in the clinical treatment of Egyptian *Staphylococcus*, but also points to the depth of knowledge still to be discovered in this research area (Abouelfetouh, 2017; Abouelfetouh et al., 2017).

In this study, we utilized genomics to report on the plasmid content of Egyptian Staphylococci, which further adds to our global knowledge of Staphylococcal plasmids. Plasmids and their contributions to antibiotic resistance have not been investigated previously within the Arab states. Putative plasmid sequences were present in over half of isolates analyzed in this study, with widespread presence of antibiotic resistance genes. Since plasmids are reservoirs of and vectors for antibiotic resistance in Staphylococci, plasmids could be a key factor in the pathology and epidemiology of Staphylococci, including MRSA and MRSH (McCarthy and Lindsay, 2012; Shahkarami et al., 2014). Making this knowledge available allows policy makers and infection control officers the chance to design and implement well-informed infection control and antimicrobial stewardship plans. Further characterization of plasmids is necessary to better understand the infectious potential of Staphylococci and devise optimal strategies to curb their spread and virulence.

## Data Availability Statement

Data analyzed as part of this study include the following GenBank WGS accession numbers: JAEOUR000000000, JAEOUZ000000000, JAEOVE000000000, JAEOVM000000000, JAEOVQ000000000, JAEOVX000000000, JAEOWM000000000, JAEOUV000000000, JAEOUX000000000, JAEOVD000000000, JAEOVF000000000, JAEOVH000000000, JAEOVJ000000000, JAEOVK000000000, JAEOVN000000000, JAEOWY000000000, JAEOVO000000000, JAEOVR000000000, JAEOVT000000000, JAEOVV000000000, JAEOVW000000000, JAEOWB000000000, JAEOWC000000000, JAEOWD000000000, JAEOWF000000000, JAEOWG000000000, JAEOWH000000000, and JAEOWK000000000.

## Author Contributions

CM, AJW, and AA designed the study. CRM and CP conducted the analyses. CRM, CM, CP, and AA wrote the initial draft. All authors contributed to the manuscript editing and approved the final version.

## Conflict of Interest

AJW discloses membership on the Advisory Boards of Pathnostics and Urobiome Therapeutics. This membership did not play a part in the design of the study, analysis or interpretation of data, writing of the manuscript, or decision to publish the results. The remaining authors declare that the research was conducted in the absence of any commercial or financial relationships that could be construed as a potential conflict of interest.

## Footnotes

1 https://sourceforge.net/projects/bbmap/

## Supplementary Material

Supplementary Table 1 Plasmid vs. genome sequence coverage.

Supplementary Table 2 BLAST-based similarity of putative plasmid sequences to records in nr/nt database.

Supplementary Table 3 Annotation of putative plasmid sequences.

Supplementary Table 4 Antibiotic resistance genes identified for each putative plasmid sequence.

Supplementary Table 5 Publicly available MLST-1 *S. aureus* genomes screened for *tet(L)*.
